# The Evolution of the Modern Hospital

**Published:** 1896-08-15

**Authors:** 


### The Evolution of the Modern Hospital.

### II.—THE LATEST HOSPITAL.

On Friday afternoon, July 31st, the Metropolitan Asylums Board afforded an opportunity, to those who had sufficient knowledge to realise the truth, by paying a visit to the Brook Hospital at Shooter's Hill, to gain an object lesson in modern hospital construction which has probably been unequalled in the history of England. This Brook Hospital is an attempt—and we may say at once a successful attempt—to apply every modern facility which science has evolved, to the treatment of infectious disease, including scarlet and enteric fevers and diphtheria. The Brook Hospital should be visited by men and women of all classes who take an intelligent interest in the progress of sociology, and who desire to realise the enormous development which has taken place in regard to the care now taken by society for the well-being of all classes in a great city like London. In the first place it is a fact worth noting that a small army of workmen have been employed for months in the erection of this vast hospital at Shooter's Hill, which will provide accommodation for their children when overtaken by infectious disease, and furnish them with every possible comfort, appliances, hygienic condition, care, and nursing which the collective wisdom of the medical and scientific worlds declare to be the best it is possible to secure. Every precaution has been taken to isolate the attendants and all who have business to do with this hospital from every avoidable risk of infection. With this view the administrative buildings have been kept separate and distinct from the wards; two entrances are provided to the hospital, and the nurses and staff when off duty will be free from the ordinary hospital surroundings.

The laundry, the water supply, the special arrangements for the protection of the buildings against fire, the heating apparatus, and the application of electricity to the whole institution, whereby lighting and telephonic communication are secured, and alarms against fire are perfected, all present features of interest and instruction. The system of receiving, sorting, disinfecting, washing, drying, ironing, and delivering the linen, from its entrance to and exit from the laundry, are well conceived, and calculated to prove successful in practice. The hardness of the water from the Kent Water Company has been overcome by providing a means of softening 60,000 gallons per day of ten hours. The heating will be carried out by central fires and a system of low pressure hot-water apparatus, with the few exceptions where the use of steam becomes essential. The hospital is lighted throughout by electricity, the current being carried to a switch-board and there distributed, and the two systems of mains are so arranged that the principal blocks and wards are supplied with current from both systems, half the lights being connected to each. The risk of extinction of the light by any accident to plant or mains is practically avoided.

The boilers and engines in connection with the warming apparatus present some ingenious and novel features. With a view to economising the consumption of all fuel, a Green's Economiser has been provided, by which the feed water, before entering the boilers, is heated up to a high temperature by utilising the waste gases from the boiler furnaces. The condense water from the greater portion of the steam heaters throughout the hospital is brought back to a condense tank close to the boiler house, from which it will be pumped up again for use in the boilers. By another ingenious contrivance and the use of Berriman's feed-water heater placed on the fire-floor of the boiler-house, the exhaust steam from the electric light engines being made to pass through this heater heats the water, which is then pumped into the boilers. Interesting as these special feature are, their practical value may be gathered from the fact that the employment of Green's economiser alone will effect a saving of not less than 15 per cent.

Turning now to the hospital proper, the expert is provided with many matters for reflection. There are twelve main ward pavilions, eight of which will be devoted to scarlet fever patients and four to enteric fever and diphtheria. These pavilions differ from those usually met with in fever hospitals, as they are all two storeys in height, and are provided with an open space of from four to eight feet in height under the ground floor. The amount of linear wall space, floor, air and cubic space is probably greater than that to be met with in all, or nearly all, existing hospitals of this class. Thus the linear wall space, floor, air and cubic space in the scarlet fever wards are respectively 12 feet, 156 feet, and 2,028 feet, and in the enteric and diphtheria wards they have been increased to 15 feet, 195 feet, and 2,535 feet respectively. Of course, the provision of so large a floor and linear space, especially in the diphtheria wards, is an experiment, and its effect upon the treatment of this dire disease will be watched with considerable interest. Whether the results will warrant the considerable extra expenditure which such prodigality of space necessitates we are inclined to doubt. On this point, and also with respect to the construction and arrangement of the isolation wards, it is no small matter that the Metropolitan Asylums Board and their advisers have had the courage to make the experiment, and to give the doctors practically a free hand so far as this provision is concerned. There are no less than six isolation pavilions, two containing four single bed wards each, and four having each one ward for four beds. Each isolation ward, whatever the bed accommodation, is entirely self-contained. The appliances, fittings, and arrangements are identical with those of the main wards, except that steam is used for heating instead of hot water. The hospital is provided throughout with ample verandah accommodation and escape staircases, the views from which are often attractive, and the architect is to be congratulated on the open ventilating fireplaces, the introduction of warmed external air by the stoves and over the copper coils through glazed channels regulated by valves. In addition there are valvular gratings in the external walls under each bed at the floor line, and windows, one to each bed, on opposite sides of the ward, which are supplemented by hopper hung fan-lights, so that an abundance of fresh external air can be admitted to the ward without difficulty. We quite agree with the architect, Mr. Aldwinckle, that mechanical ventilation is entirely unsuitable to this country, where on about 300 to 330 days in the year it is possible to open the windows of a hospital ward without injury to the patients.

